# Supplementary material for: Blue Light-Induced Mitochondrial Oxidative Damage Underlay Retinal Pigment Epithelial Cell Apoptosis
Source: Int J Mol Sci. 2024 Nov 24;25(23):12619. doi: 10.3390/ijms252312619 (PMC11641757; doi:10.3390/ijms252312619)
Supplement: Supplementary file 1 [file ijms-25-12619-s001.zip › ijms-3295894-supplementary.pdf]

Blue light-induced mitochondrial oxidative damage underlay retinal pigment epithelial cell apoptosis

Mohamed Abdouh[a,b], Yunxi Chen[a], Alicia Goyeneche[a,b], Miguel N. Burnier[a,b]

[a] Cancer Research Program, Research Institute of the McGill University Health Centre, Montreal, QC, Canada. [b] The MUHC-McGill University Ocular Pathology & Translational Research Laboratory, Montreal, QC, Canada

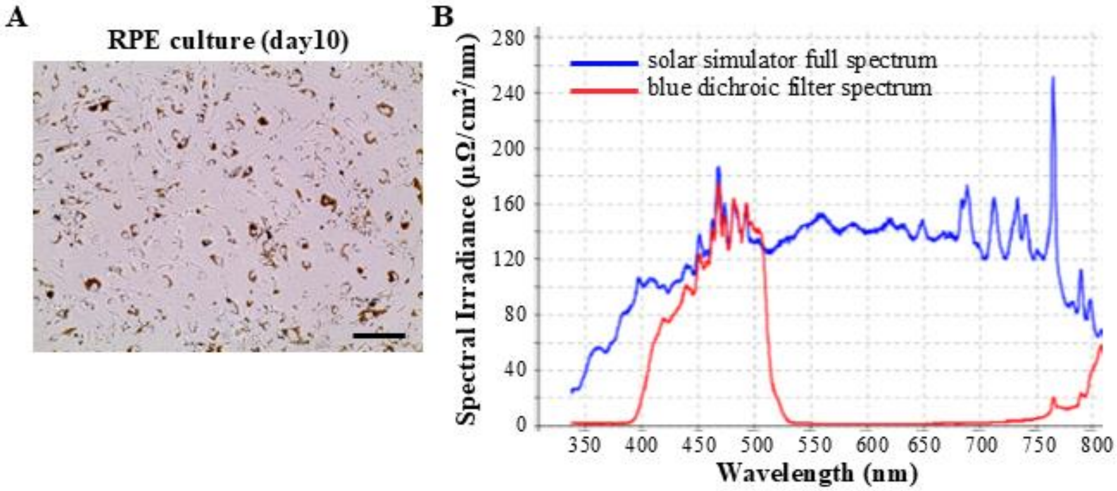

Figure S1. Primary human RPE cell culture and representative light spectra. (A) Primary human RPE cell cultures were established from donor eyes as reported previously (34, 82). During the first 1 to 4 passages, cells displayed a brown-dark pigmented feature. Scale bars: 100 µm. **(B)** Transmission spectra of the solar simulator without (blue line) or with (red line) the application of the blue dichroic filter. Measurements were made using a *STS-VIS* spectrometer (Ocean Optics).

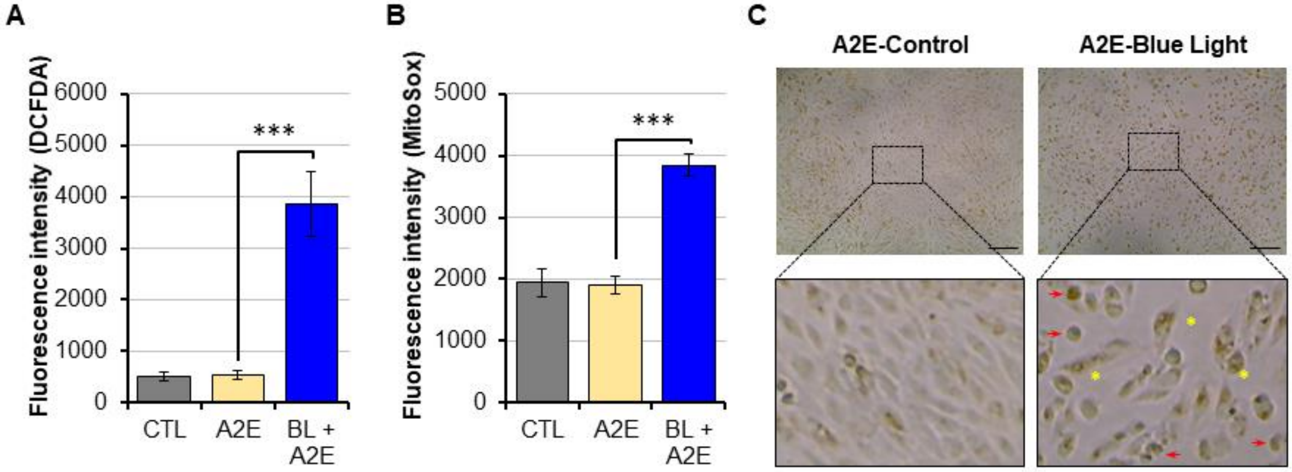

Figure S2. BL induced oxidative stress in A2E-loaded ARPE19 cells. ARPE19 cells were exposed to BL for 30 min. **(A)** Cells were analyzed for the production of total cellular ROS using the DCF-DA probe. **(B)** Cells were analyzed for the production of mitochondrial superoxide anion using the MitoSox Red probe. Data are presented as mean +/- SD (n = 3 independent experiments each repeated in quadruplicates, ***P < 0.001). **(C)** Phase contrast views of cells without (A2E-Control) and 48 hours following exposure to blue light (A2E-Blue Light). Note that cells exposed to BL shrunk (red arrowheads) and detached from the plate leaving cell-free spots (yellow stars). Scale bar: 40 µm.

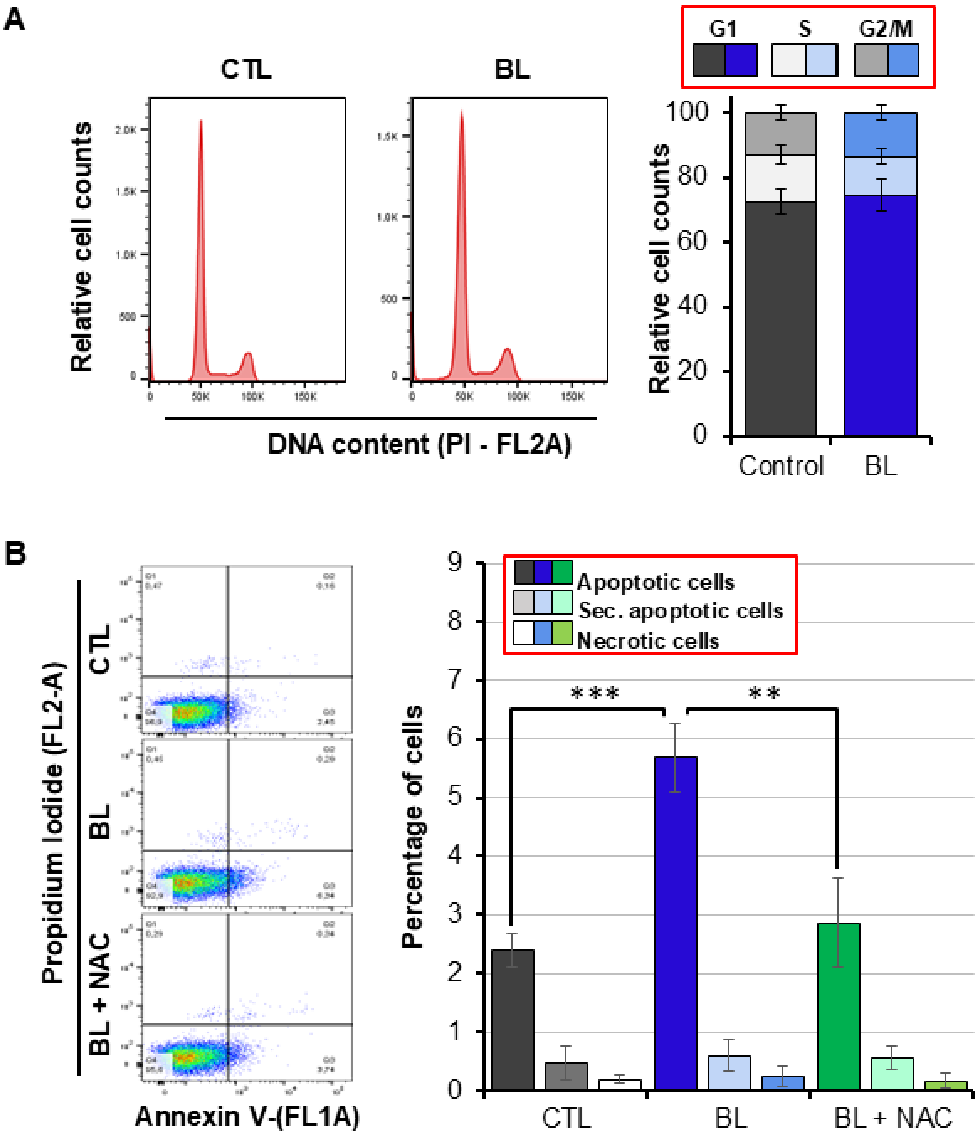

Figure S3. BL induced RPE cells apoptosis in ROS-dependent manner. ARPE19 cells were exposed to BL for 30 min. **(A)** 24 hrs post-BL exposure, cells were labeled with propidium iodide (PI) and analyzed for their proliferation. Representative cell cycle phase distribution histograms are shown, and the graph displays the percentages of cells in the different phases of cell cycle. (**B**) 6 hrs post-BL exposure, cells were labeled with Annexin V and PI and analyzed for the percentages of apoptotic cells by flow cytometry. Representative Annexin V/PI density plots are shown, and the graph displays the percentages of primary apoptotic, secondary apoptotic and necrotic cells. Data are presented as mean +/- SD (n = 4 independent experiments, $^{**}P < 0.01$, $^{***}P < 0.001$).

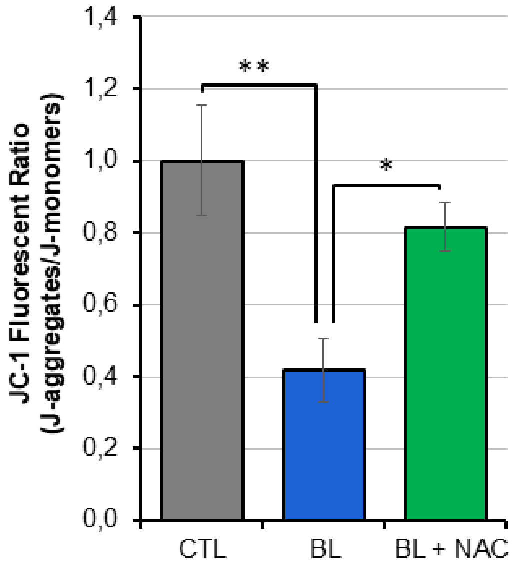

Figure S4. BL reduced the mitochondrial membrane potential in ROS-dependent manner. ARPE19 cells were exposed to BL for 30 min, and cells were stained with JC-1 probe. Fluorescence of J-aggregates and J-monomers were measured. Data are expressed as the ratio between the 2 measures and are presented as mean +/- SD (n = 7 independent experiments each repeated in quadruplicates, * P < 0.05, **P < 0.01.

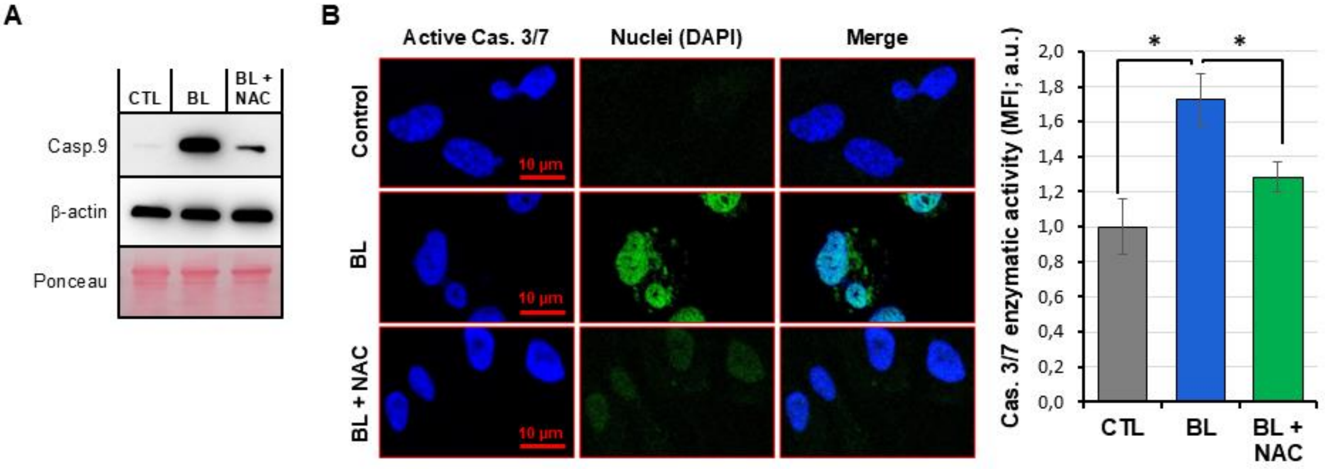

Figure S5. BL increased caspase cascade activation in ROS-dependent manner. ARPE19 cells were exposed to BL for 30 min. **(A)** Proteins extracts were analyzed by immunoblot for the activation of Caspase 9. β-actin and red ponceau staining were used as calibrators for proteins loading. **(B)** Cells were loaded with CellEvent Caspase 3/7 Green. Pictures were acquired using a LSM780 confocal microscope. The graph displays the levels of caspases 3 and 7 activation in the corresponding samples. Data are expressed as mean fluorescence intensity (MFI) measured in an Infinite M200Pro microplate reader relative to the value in control sample set at 1. Data are presented as mean +/- SD (n = 2 - 4 independent experiments), * P < 0.05.

Supplementary Table Legends

Table S1. Proteomic analyses identified 2810 proteins (of which 1404 were detected in all analysed RPE samples).

Table S2. Proteins highly expressed in control non-exposed RPE cells

Table S3. Proteins highly expressed in BL-exposed RPE cells

Table S4. Proteins involved in the cellular response to oxidative stress
